# Reactive Oxygen Species in Drought-Induced Stomatal Closure: The Potential Roles of NPR1

**DOI:** 10.3390/plants12183194

**Published:** 2023-09-07

**Authors:** Xin-Cheng Li, Claire Chang, Zhen-Ming Pei

**Affiliations:** 1 East Chapel Hill High School, 500 Weaver Dairy Rd, Chapel Hill, NC 27514, USA; clairechang604@gmail.com; 2 Department of Biology, Duke University, Durham, NC 27708, USA; zpei@duke.edu

**Keywords:** drought, stress response, stomatal closure, reactive oxygen species, cell death, antioxidants, salicylic acid, NPR1

## Abstract

Stomatal closure is a vital, adaptive mechanism that plants utilize to minimize water loss and withstand drought conditions. We will briefly review the pathway triggered by drought that governs stomatal closure, with specific focuses on salicylic acid (SA) and reactive oxygen species (ROS). We propose that the non-expressor of PR Gene 1 (NPR1), a protein that protects plants during pathogen infections, also responds to SA during drought to sustain ROS levels and prevent ROS-induced cell death. We will examine the evidence underpinning this hypothesis and discuss potential strategies for its practical implementation.

## 1. Introduction

Drought stress is a major abiotic constraint that adversely affects plant growth, development, and crop productivity worldwide. To withstand water scarcity, plants have evolved various physiological and molecular mechanisms to regulate water loss and maintain cellular homeostasis [1]. Among these mechanisms, stomatal closure plays a pivotal role in reducing transpiration water loss through the regulation of gas exchange [2]. Stomata, microscopic pores on leaf surfaces, are controlled by a complex signaling pathway that integrates environmental cues [3] and internal hormonal signals such as salicylic acid (SA) [4]. This paper aims to provide a comprehensive overview of the stomatal closure pathway in response to drought stress, focusing on the underlying molecular and physiological processes that involve reactive oxygen species (ROS), antioxidants, and SA.

## 2. ROS Responses in Plants

One of the unavoidable results of drought stress is increased ROS production in plants [5]. ROS are highly reactive molecules that are formed as byproducts of the metabolism of oxygen. There are many different types of ROS, including hydrogen peroxide (H_2_O_2_), singlet oxygen (^1^O_2_), superoxide (O_2_^−^·), and the hydroxyl radical (HO^•^). Each type of ROS reacts within cells differently; superoxide usually reacts with other molecules to form secondary oxidants [6], hydroxyl radicals can damage DNA [7], hydrogen peroxide mainly reacts with cysteine residues of proteins [8], and singlet oxygen can react with both proteins [9] and DNA [10].

In plants, ROS can be generated through multiple different pathways. Under physiological conditions in chloroplasts, the splitting of water at Photosystem II (PSII) during the light-dependent reactions of photosynthesis can form superoxide and singlet oxygen [11]. Superoxide is also formed by Complex I and Complex III in the mitochondrial electron transport chain during the formation of ubisemiquinone [12]. From superoxide, hydrogen peroxide can be formed via superoxide dismutase (SOD) [13]. Under stress conditions, ROS can be synthesized by cell wall peroxidases and NADPH oxidase [14].

ROS can function as secondary messenger molecules during signal transduction processes [15]. In such instances, hydrogen peroxide oxidizes target proteins at their cysteine residues, leading to alterations in protein structure and function [16]. One intrinsic limitation of ROS, however, is that, due to their high reactivity, in high concentrations, ROS can cause irreversible cell damage or even cell death. In the presence of biotic and abiotic stressors, ROS increases the permeability of mitochondria, leading to the release of cytochrome C (Cyt C) to the cytosol. The loss of Cyt C subsequently impairs the functionality of the mitochondrial electron transport chain, exacerbating the buildup of ROS. This creates a self-perpetuating cycle of ROS accumulation, thereby intensifying the positive feedback loop triggering programmed cell death (PCD) [17]. To prevent ROS-induced cell death, ROS are regulated by antioxidants, which include chemicals such as flavonoids [18] and carotenoids [19], and proteins such as catalase (CAT) and glutathione peroxidase (GPX) [20]. These antioxidants neutralize ROS by transferring electrons, often eventually converting ROS into water [21].

## 3. ROS and Hormone Crosstalk in Stomatal Closure

ROS are directly implicated in the process of drought-induced stomatal closure [22]. This intricate process, which occurs within guard cells, is regulated by various plant hormones, including abscisic acid (ABA) and salicylic acid (SA) [3]. When confronted with drought stress, plants generate ABA in their roots [23,24,25], where drought-induced turgor loss is detected, and transmit the ABA signal to leaves through the xylem. ABA is derived from a C_40_ carotenoid precursor, which is cleaved into the intermediate xanthoxin and then converted into ABA. ABA binds to its receptor PYR/PYL [26] and induces the recruitment and inhibition of PP2Cs [27], a group of phosphatases that dephosphorylate and deactivate the protein kinase OST1 [28]. The inhibition of PP2Cs leaves OST1 phosphorylated, enabling it to phosphorylate Ser120 of SLAC1 anion channels. However, the phosphorylation of Ser120 alone is not sufficient to activate SLAC1 [29]. At this juncture, ROS comes into play. Besides directly phosphorylating SLAC1, OST1 phosphorylates the NADPH oxidase RBOHF [30], which subsequently produces superoxide that is converted by superoxide dismutase (SOD) into hydrogen peroxide [31]. Hydrogen peroxide then activates calcium channels on the plasma membrane of guard cells [32] through the oxidation of extracellular cysteine residues on HPCA1 kinase, leading to autophosphorylation and the subsequent activation of the calcium channels, causing calcium influx [33]. The increased calcium induces calcium-dependent protein kinases (CPKs) to phosphorylate Ser59 of SLAC1. Only when both serines, Ser120 and Ser 59, are phosphorylated does SLAC1 initiate anion efflux from the cell [34,35]. The efflux of anions from the guard cell creates a concentration gradient, compelling water to exit the cell to restore equilibrium, thus causing a decrease in cell volume and leading to stomatal closure [36]. While most of these studies were conducted in *Arabidopsis thaliana*, in rice, hydrogen peroxide production also experiences a dramatic increase upon ABA induction. Following the calcium influx induced by ABA, Ca^2+^/calmodulin-dependent protein kinase (DMI3) phosphorylates Ser191 of RBOHB, leading to superoxide production. The superoxide flux then activates channels permitting Ca^2+^ to flow into the cytosol. This Ca^2+^ influx, in turn, further induces OsDMI_3_-mediated phosphorylation, which further intensifies superoxide production during the ABA response, creating a positive feedback loop [37,38].

ABA is not the sole hormone to induce ROS production during stomata closure. Under drought conditions, jasmonic acid (JA) assumes the form of JA-isoleucine (JA-Ile), which relocates to the nucleus and activates the coronatine insensitive 1 (COI1) protein within the Skp1p–cullin–F-box protein (SCF) E3 ligase complex, as well as a range of transcription factors [39]. Under physiological conditions, these transcription factors are sequestered from DNA by jasmonate-zim-domain (JAZ) proteins. Upon JA-Ile engagement, the JAZ proteins are degraded by SCF-COI1 and the 26S proteasome. This liberates transcription factors and activates an array of genes responsible for the JA response [40]. In *Hevea brasiliensis*, the presence of JA leads to the upregulation of the transcription of RBOHF, aiding in the generation of ROS [41]. 

**Figure 1:**
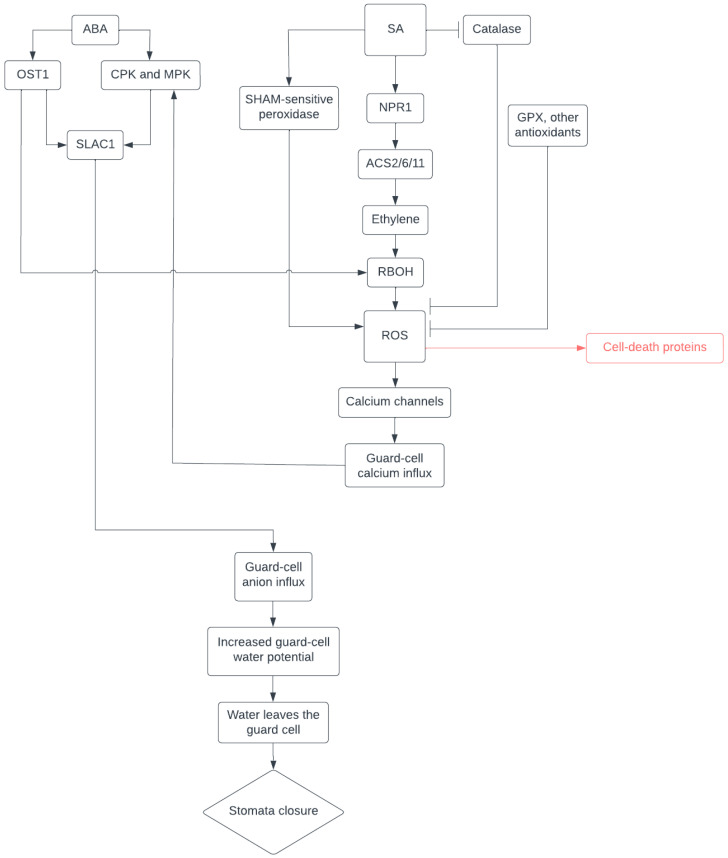
Current working model of stomatal closure.

Similarly, SA, another hormone, also contributes to drought-induced stomatal closure through the activation of ROS generating enzymes (Figure 1). Drought stress prompts the heightened expression of isochorismate synthase 1 (ICS1), a gene essential for SA biosynthesis [42,43]. The accumulation of SA has been observed to both augment drought tolerance [44,45] and induce stomatal closure [46,47]. Specifically, SA contributes to stomatal closure by fostering ROS accumulation [46]. During stomatal closure, SA induces SHAM-sensitive peroxidases to produce superoxide. The proposed mechanism is that SA reduces Compound I and II of peroxidase, forming SA free radical species that react with oxygen gas to create superoxide and SA+ [48,49]. The resulting superoxide is converted into hydrogen peroxide via SOD [22,46,49]. Simultaneously, SA inhibits the antioxidant catalase, further increasing ROS levels [50,51]. The ROS generated by SA integrates into the ABA-induced stomatal closure pathway, activating calcium channels and instigating calcium influx [36].

While both SA and JA have been demonstrated to induce ROS production in response to various stress conditions, intriguingly, their interplay does not exhibit synergy; rather, it displays antagonism. Particularly under pathogen-induced stress, JA prompts the transcription factor MYC2 to directly bind to the promoters of multiple NAC transcription factors, thereby activating their transcription. These NAC transcription factors subsequently restrain the expression of ICS1, a key enzyme in SA biosynthesis [52]. Some proteins such as UGT76B1 (Table 1) have also been shown to decrease SA response while increasing JA response. Conversely, SA triggers the upregulation of ROXY19/GRX480, which impedes the TGA transcription factors from facilitating the expression of JA response genes [53]. The biological subtlety from this complex crosstalk remains elusive, particularly in the context of drought stress. 

While it is recognized that SA competitively inhibits antioxidant catalase-2 (CAT2) as an antagonist [50], numerous other antioxidants, such as GPX, are known to be regulated by SA during stomatal closure with elusive mechanisms. Another unsolved puzzle is how guard cells avoid cell death under high ROS levels induced by SA and ABA. However, potential clues to these questions can be found in the known roles of SA in managing other stress responses.

## 4. SA Is a Key Modulator in General Stress Response

SA is a phenolic compound naturally present in plants. SA biosynthesis is tightly regulated and can proceed via several different pathways, with the phenylpropanoid and isochorismate pathways being the most prevalent [80]. In the phenylpropanoid pathway, the amino acid phenylalanine is converted into SA by phenylalanine ammonia lyase, while in the isochorismate pathway, isochorismate synthase converts chorismate into isochorismate which then acts as a precursor to SA [81]. SA serves as a key signaling molecule within various physiological and pathological processes and is primarily known for its role in regulating defensive mechanisms in plant abiotic stress response pathways and pathogen immunity [82].

During a pathogen attack, the plant initiates a series of signaling events that lead to an increase in SA levels. A primary consequence of this SA accumulation is the activation of defense-related genes. SA acts as a ligand for NPR1 (Non-expressor of Pathogenesis-Related Genes 1), leading to the activation of downstream defense genes. These genes produce various antimicrobial compounds, including pathogenesis-related (PR) proteins that bind to pathogens and inhibit their growth [83].

In response to pathogen invasion, particularly as effector-triggered immunity (ETI) in plants, apoptosis can be activated in cells neighboring the infection site to isolate the pathogen. However, if left unregulated, ETI-associated apoptosis can spread, leading to excessive levels of programmed cell death (PCD). SA-induced NPR1 can counteract potential spread by conjugating to form salicylic-acid-induced NPR1 condensates (SINCs) within the cytoplasm. This condensate assembles the NPR1-associated proteins with Cullin 3 E3 ligase complex, which marks PCD proteins such as EDS1 and WRKYs for ubiquitination and subsequent protein degradation, thereby promoting cell survival [84]. This mechanism of promoting cell survival is not exclusive to pathogen stress; both SA and NPR1 are required to support cell survival under other stressors, including heat, oxidative stress, and DNA damage. This suggests that SA and NPR1 play pivotal roles in coordinating stress response and cell survival. 

## 5. NPR1 May Coordinate Comprehensive Protection during Drought Stress 

The formation of SA-induced NPR1 condensates may also be essential for stomatal closure and cell survival during drought stress. It has, indeed, been noted that NPR1 expression increases in response to drought, and this increase has been implicated in promoting ROS generation and stomatal closure [42,85]. Upon SA increase, NPR1-activated gene expression includes ACS2/6/11, enzymes for ethylene precursor synthesis. Ethylene, in turn, promotes ROS production by activating ATRBOHD, an NADPH oxidase. The disruption of ethylene production through the mutation of ethylene biosynthesis genes inhibits SA-induced stomatal closure, suggesting that ethylene-induced ROS production is necessary for this process [85]. 

Furthermore, our literature review revealed that, during pathogen stress, NPR1 condensates triggered by SA also incorporate various proteins that potentially contribute to sustaining ROS responses. In the proteomics data published in an article by Dong and colleagues, which studies NPR1’s response against pathogens [84], we identified six proteins directly involved in ROS scavenging, an additional five proteins participating in antioxidant biosynthesis, and seven proteins that assist other antioxidants in ROS scavenging (Table 1). Among these proteins are Glutathione Peroxidase 8 (GPX8), which has been shown to have decreased expression during drought [56], and selenoprotein, the absence of which has been linked to increased drought tolerance [57]. Given these proteins’ unilateral roles in ROS scavenging, their degradation by NPR1 would lead to an accumulation of ROS, thereby inducing stomatal closure and enhancing drought tolerance.

Notablt, during pathogen infection, GSTU19—a member of the GST family—is detected within NPR1-associated complexes, implying potential degradation. GSTU19 is well-known for its role in catalyzing the degradation of ROS, primarily hydrogen peroxide [86]. Such degradation of GSTU19 could result in increased ROS levels. Yet, under drought conditions, studies have observed an upregulation of GSTU19 at both the mRNA and protein levels in Manihot esculenta [87]. This raises two plausible scenarios: (1) NPR1 may not target or degrade GSTU19 during a drought response; or (2) even if NPR1 condensate degrades GSTU19 during drought, the degradation might not be robust enough to offset its amplified expression. Currently, the exact dynamics remain unclear. The regulation of GSTU19 underscores the nuanced control of ROS during drought responses.

**Figure 2:**
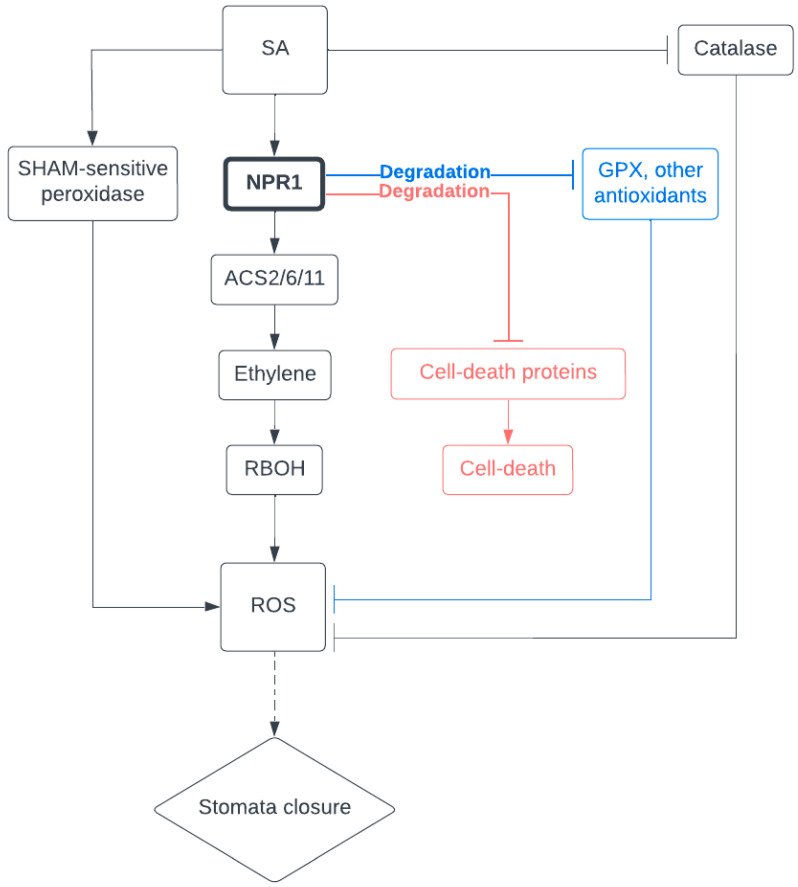
NPR1, a potential crucial regulator of stomatal closure.

Taking this together, we hypothesize that, in stomatal closure, SA induces NPR1 to form condensates with antioxidant proteins and precursors and tag them for degradation via the 26S proteasome. Along with the other functions of SA during stomatal closure, such as the activation of ROS-generating proteins, the inhibition of ROS-degrading proteins may be crucial in maintaining cellular ROS at an elevated level, and therefore sustain the stomatal closure. Meanwhile, SA will also induce NPR1 to form condensates to degrade multiple key cell death proteins, preventing guard cells from programmed death under the constant ROS stress (Figure 2).

## 6. Perspectives

Research indicates that NPR1 is essential for cell survival during drought stress. Enhancing the expression of NPR1 in plants might be a potential strategy to boost plant resistance to drought. However, the perpetual overexpression of NPR1 has been associated, in some cases, with decreased plant growth and vitality [88]. To circumvent these fitness costs, in a 2017 study Xu et al. utilized upstream open reading frames (uORFs) to suppress the translation of NPR1 under normal conditions. Their findings showed that these genetically modified plants could grow normally while also exhibiting enhanced pathogen resistance due to the stress-induced overexpression of NPR1 [89]. Utilizing the same molecular biology maneuver could be an effective approach to improve drought resistance. Future experiments are needed to determine whether overexpressing NPR1 can prevent plants from death in drought, and whether drought stress is able to overcome uORF inhibition. 

## Figures and Tables

**Table 1:** ROS-related proteins identified in NPR1 condensates [51].

	Protein Discovered in NPR1 Condensate Proteomics Study [51]	Explanation of Protein Function	*p*-Value in Discovery
Direct ROS scavengers	OPR1	Neutralizes ROS [54]	0.000052
	ALDH7B4	Involved in detoxification and is involved in reducing oxidative stress [55]	0.005
	GPX8	Converts H_2_O_2_ to H_2_O [56]	0.007
	Selenoprotein family protein	Involved in breaking down H_2_O_2_ to H_2_O [57,58]	0.04
	Aldolase-type TIM barrel family protein	Protects against H_2_O_2_, suggests that they may be involved in scavenging H_2_O_2_ [59]	0.042
	Thioredoxin superfamily protein	Scavenges ROS [60]	0.044
Positive regulators of ROS scavenger biosynthesis	DMR6	Has flavone synthase activity. Flavones directly decrease the amount of ROS [61,62]	0.000025
	CYP51	Involved in sterol biosynthesis. Sterol can serve as an ROS scavenger [63,64]	0.006
	SQE3	Required for sterol biosynthesis. Sterols can serve as ROS scavengers [65]	0.014
	Thioesterase superfamily protein	Vitamin K biosynthesis requires thioesterases. The Vitamin K cycle has antioxidant activity [66,67]	0.031
	ATR4, CYP83B1, RED1, RNT1, SUR2, cytochrome P450, family 83, subfamily B, polypeptide 1	Involved in glucosinolate biosynthesis. Glucosinolate decreases ROS levels [68,69]	0.046
Proteins facilitating ROS neutralization	GRF6	ANKR2A-APX3 complex is a protein complex that degrades H_2_O_2_, and GRF6 is found to interact with the complex during antioxidant defense [70,71]	0.002
	GSTU19	Interacts with the protein GPX when GPX breaks down ROS [72]	0.002
	UGT73B2	Glycosylates quercetin, which is a flavonoid that reduces H_2_O_2_ to H_2_O [73]	0.003
	ATMDAR2	Involved in the ascorbate–glutathione cycle, which serves to break down H_2_O_2_ to H_2_O [74]	0.012
	GDH1	GPX is a protein that converts H_2_O_2_ to H_2_O. GPX requires GDH1 to function [75]	0.016
	G6PD6	Reduces ROS under redox stress by supplying NADPH [76,77]	0.035
	Zim-17 type zinc finger protein	Essential for facilitating zinc binding. Zinc acts as a cofactor for reducing ROS [78]	0.04
Hormone crosstalk	UDP-Glycosyltransfersae superfamily protein (UGT76B1)	Reduces SA response and promotes JA response [79]	0.04

## Data Availability

The data discussed in this review are shown in previously published studies.
